# *Aspergillus awamori* positively impacts the growth performance, nutrient digestibility, antioxidative activity and immune responses of growing rabbits

**DOI:** 10.1002/vms3.345

**Published:** 2020-09-09

**Authors:** Mahmoud H. El‐Deep, Mahmoud A. O. Dawood, Mohamed H. Assar, Bilal Ahamad Paray

**Affiliations:** ^1^ Animal Production Research Institute Sakha Station Kafr El‐Sheikh Egypt; ^2^ Department of Animal Production Faculty of Agriculture Kafrelsheikh University Kafr El‐Sheikh Egypt; ^3^ Department of Zoology College of Science King Saud University Riyadh ‐ 11451 Kingdom of Saudi Arabia

**Keywords:** feed utilization, growing rabbits, health condition, histomorphology, probiotics

## Abstract

In this study, we explored the effects of dietary administration of *Aspergillus awamori* on the growth, intestinal histomorphology, immune parameters and antioxidant enzyme activity of growing rabbits. The rabbits of 5 weeks of age (body weight, 855 ± 5.53 g) were allotted into four groups (12 rabbits per group) and fed four experimental diets enriched with *A. awamori* at 0, 50, 100 and 150 mg per kg diet for 8 weeks. After the feeding trial, an equal number of male APRI rabbits (3 months old; 4 groups, 5 rabbits per group) were housed in metabolism cages and fed the test diets for 14 days for the digestibility trial. All *A. awamori*‐enriched diets induced a significant increase in the average body weight and weight gain of rabbits and significantly decreased the feed conversion ratio. In rabbits fed with *A. awamori* at 100 or 150 mg per kg diet, protein, lipid and fibber digestibility coefficients significantly increased compared with the control group. Intestinal villi measurements (length and thickness) were also enhanced in all rabbits fed with *A. awamori*. Adding *A. awamori* as a food supplement generally did not affect rabbit haematology and blood biochemistry values; however, at 150 mg per kg diet, it significantly increased the levels of haemoglobin and total protein, as well as red blood cell count. Furthermore, all enriched diets significantly increased rabbits’ phagocytic activity and their phagocytic index. Rabbits fed with *A. awamori* also showed decreased malondialdehyde but increased catalase activity. In conclusion, *A. awamori* administered as feed supplement at 100–150 mg per kg of growing rabbits’ diet enhances their growth, intestinal health and nutrient digestibility, and it raises the levels of their immune and antioxidative responses.

## Graphical abstract

In this study, we have explored the effect of dietary administration of Aspergillus awamori on growth, intestinal histomorphology, immune parameters and antioxidant enzyme activity of growing rabbits.

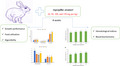

## 1 INTRODUCTION

Infectious disease is one of the major limiting factors for the expansion of the rabbit industry. Intensified cultures, in which rabbits are reared at high densities, facilitate the spread of pathogens and the occurrence of disease outbreaks due to stressed and immunosuppressed animals (Licois, Coudert, Ceré, & Vautherot, 2000). The use of veterinary drugs for the treatment of infectious diseases often has health and environmental side‐effects; thus, alternative, more sustainable, prevention and treatment strategies are needed to ensure expected growth (Bovera et al., 2010). The use of feed supplements with immunostimulant properties (e.g., medicinal plants or probiotics) has gained popularity in recent decades (Barton, 2000; Markowiak & Śliżewska, 2018). Using probiotic‐enriched diets is an inexpensive practice that can be adopted by both small‐ and large‐scale rabbit farms; this practice offers several benefits from increasing rabbit growth to increasing immune parameters and disease resistance (Ayyat, Al‐Sagheer, Abd El‐Latif, & Khalil, 2018). Indeed, the use of probiotics has increased due to their remarkable beneficial effects on microbiota and gut health in swine (Lessard et al., 2009), poultry (Saleh et al., 2012) and rabbits (Bhatt, Agrawal, & Sahoo, 2017; Copeland, McVay, Dassinger, Jackson, & Smith, 2009; Phuoc & Jamikorn, 2017).

In food processing, *Aspergillus* spp. are used to ferment food components and increase nutritional value (Gawande & Kamat, 1999). *Aspergillus* spp. improves the growth performance and well‐being of its host due to the capacity of these fungi to stimulate immune responses and inhibit the growth of pathogenic bacteria by reducing pH levels in the gut (Bhatt et al., 2017; Copeland et al., 2009; Phuoc & Jamikorn, 2017). *Aspergillus* spp. also generate antibacterial components, including organic acids, bacteriocins, hydrogen peroxide, diacetyl, acetaldehyde, the lactoperoxidase system and lactones, which have a positive impact on local intestinal immunity as well as the diversity of microbiota (Tamang, Shin, Jung, & Chae, 2016; Zubillaga et al., 2001). Given these benefits, the Food and Drug Administration has categorized *Aspergillus awamori* as a unique strain of probiotic approved for administration in animals (Saleh et al., 2012; Saleh, Hayashi, Ijiri, & Ohtsuka, 2015). Indeed, dietary *A. awamori* supplementation has been shown to improve growth performance and feed efficiency in animals due to the production of vitamins and the secretion of amylase, protease and lipase, which can increase the digestibility of nutrients in an animal's gut (Mahmoud H et al., 2013; Saleh, Amber, et al., 2014; Saleh et al., 2011; Zubillaga et al., 2001).

In the present study, for the first time, we investigated the effects of dietary supplementation with *A. awamori* on the growth performance, feed digestibility, immune response and oxidative status of growing rabbits.

## 2 MATERIALS AND METHODS

### 2.1 Source of A. awamori

*A. awamori* (25 × 10^4^ cells per g) was provided in powder form from Biogenkoji Research Institute, Kirishima, Japan.

### 2.2 Rabbits, diets and experimental procedure

The experimental procedure was approved by the Faculty of Agriculture, Kafrelsheikh University, Egypt (07–2018). The study was investigated by performing a feeding trial for eight weeks, followed by a digestibility trial for 14 days. In total, 48 APRI line weanling rabbits at 5 weeks of age were obtained from the Animal Production Research Institute, Sakha Station, Kafr El‐Sheikh, Egypt, and divided randomly into four groups (12 rabbits per group: six males + six females). All weanling rabbits were approximately equal in live body weight at the beginning of the experiment (855 ± 5.53 g).

The basal diet (17.4% crude protein and 2,611 kcal energy content) was daily ground to guarantee the viability of *A. awamori* to pass through a 5‐mm sieve (Table 1). All treatment diets were daily prepared by mixing the basal diet with probiotic powders in the presence of molasses to hold the *A. awamori* to feed. Four experimental diets were mixed with *A. awamori* (25 × 10^4^ cells per g) (Saleh et al., 2015) at a rate of 0 (control), 50, 100 and 150 mg per kg diet. Essential amino acids, lysine and sulphur amino acids, in addition to minerals and vitamins, were adjusted in all diets to cover the requirements according to De Bias and Mateos (2010). The digestible energy (DE) was calculated following the methods of NRC (1977) and Fekete (1986) using the following equation:

$$\text{DE}\left(\text{kcal}/\text{kg DM}\right)=4253‐32.6\times \text{Crude fibre}\left(\%\;\text{DM}\right)‐144.4\times \text{Ash}\left(\%\;\text{DM}\right)$$

**TABLE 1:** Composition and chemical analysis of the basal diet

Ingredients	%	Chemical analysis (% as dry matter)	%
Berseem hay	30.05	Dry matter	85.81
Barley grain	24.60	Crude protein	17.36
Wheat bran	21.50	Organic matter	91.42
Soybean meal (44%)	17.50	Crude fibre	12.37
Molasses	3.00	Ether extract	2.23
Limestone	0.95	Digestible energy (kcal/kg)^b^	2,610.79
Di‐calcium phosphate	1.60	Calcium	1.243
Sodium chloride	0.30	Total phosphorus	0.81
Mineral‐vitamin premix^a^	0.30	Methionine	0.45
DL‐Methionine	0.20	Lysine	0.86

^a^ One kilogramme of mineral–vitamin premix provided: Vitamin A, 150,000 UI; Vitamin E, 100 mg; Vitamin K_3_, 21 mg; Vitamin B_1_, 10 mg; VitaminB_2_, 40 mg; Vitamin B_6_, 15 mg; Pantothenic acid, 100 mg; Vitamin B_12_, 0.1 mg; Niacin, 200 mg; Folic acid, 10 mg; Biotin, 0.5 mg; Choline chloride, 5,000 mg; Fe, 0.3 mg; Mn, 600 mg; Cu, 50 mg; Co, 2 mg; Se, 1 mg and Zn, 450 mg.

^b^ Calculated according to NRC (1997) and Fekete and Gippert (1986) as: Digestible energy (kcal/kg DM) = 4253–32.6 × Crude fibre (% DM)–144.4 × Ash (% DM).

Rabbits with the same weight were kept in batteries (60 × 50 × 35 cm). Feed and water were added ad libitum from 5 to 13 weeks of age via fodder and automatic nipple drinkers. The rabbits were subjected to a 16:8 hr light: dark photoperiod regime. Weight gain and feed conversion ratio parameters were calculated according to the following formulae:

Weight gain (g) = W2‐W1, Feed conversion ratio = weight of feed consumed (g)/ body weight gain (g), where W1 is the initial weight and W2 is the final body weight.

### 2.3 Final sampling

At the end of the growing period, six rabbits (three males + three females) of 13 weeks of age were randomly selected, anesthetized (isoflurane, 5 mg/kg) (Parasuraman, Raveendran, & Kesavan, 2010) and blood samples were collected. The blood was collected from the lateral ear vein of slaughtered rabbits (5 rabbits each group), where EDTA (Ethylenediaminetetraacetic acid) was used as an anticoagulant (1 mg/ml) to collect the first aliquot for haematological parameters. Heparinized syringes were used to collect blood for total erythrocytes (red blood cell count: RBC) and total leukocytes (white blood cell count: WBC) according to the method of Reitman and Frankel (1957). The packed cell volume (PVC) was estimated using the microhematocrit method. Haemoglobin concentration (Hb) was measured using the cyanmethemoglobin method. Subsequently, the mean corpuscular volume [MCV = (PCV × 10)/ RBC]c, mean cell haemoglobin [MCH = (Hb × 10)/ RBC] and mean corpuscular Hb concentration [MCHC = (Hb × 10)/ PCV] were calculated. Blood smears were used to determine heterophils, lymphocytes, monocytes, basophils and eosinophils, as well as the heterophil: lymphocyte (H:L) ratio. Blood was collected using non‐heparinized syringes for serum separation (1,008 *g* for 15 min at 4°C), and then maintained at −20°C prior to biochemical analysis. Total protein, albumin, glucose, catalase (CAT), urea, creatinine and malonaldehyde (MDA) were determined using commercial kits (Diamond Diagnostic, Dokki, Giza, Egypt) according to the manufacturer's instructions. Serum activities of aspartate aminotransferase and alanine aminotransferase were determined (Natt & Herrick, 1952). Globulin concentration was obtained by subtracting the value of albumin from the corresponding value of total protein.

### 2.4 Digestibility trial

An equal number of male APRI rabbits (3 months old; 4 groups, 5 rabbits per group) were housed in metabolism cages and fed the test diets for 14 days for the digestibility trial and faeces were collected for 4 days as a collection period (Perez et al., 1995). Faeces were sampled in polyethylene bags and maintained at −20ºC prior to further analysis. Chemical analysis was carried out for diets, and hard and soft faeces samples, according to the methods of AOAC (2007) to assess for ash, crude protein, crude fibres and total lipids. Digestibility coefficients were calculated using the method described by Safwat, Sarmiento‐Franco, Santos‐Ricalde, Nieves, and Sandoval‐Castro (2015).

### 2.5 Immunological parameters

Phagocytosis of polymorphonuclear cells using *Candida albicans* was performed according to the methods of Rudkin et al. (2013). In a plastic tube, the following aliquots were mixed: 100 µl of fetal calf serum, 100 µl of heat‐killed *C. albicans* (5 × 10^6^ cells per ml) and 100 µl of blood. The tubes were mixed and incubated at 37°C for 30 min, after which the mixture was centrifuged at 112 *g* for 5 min. The supernatant was discarded leaving a droplet into which the sediment was re‐suspended. Smears were prepared from the deposit, dried in the air, fixed with methyl alcohol and stained with Giemsa stain. In total, 100 heterophils were examined and the number of heterophils ingesting *Candida* was counted and expressed as a percentage.

### 2.6 Morphometric analysis

For microscopic studies, samples from the small intestines of three rabbits per group were removed immediately after the rabbits had been euthanized by using 10% neutral formalin. After fixation, portions of tissue were dehydrated in increasing ethanol concentrations, cleared in xylene, embedded in paraffin blocks and sectioned to a thickness of ~5 μm. The sections were then deparaffinized with xylene and rehydrated in decreasing concentrations of ethanol. Finally, they were stained with haematoxylin and eosin for histopathological examination (Bancroft & Gamble, 2008). Morphological parameters were measured using the Image Pro Plus v 4.5 software package according to the methods of Sakamoto et al. (2000).

### 2.7 Statistical analysis

Data were analysed using SPSS software version 22. Significant differences between treatments were determined using a one‐way ANOVA followed by Duncan's multiple‐range test and defined as *p* < .05. Before analysing data, the assumptions of normality and homogeneity of variance were confirmed using the Shapiro–Wilk test and Levene's test respectively.

## 3 RESULTS

### 3.1 Growth performance

Rabbits fed with a diet enriched with *A. awamori* at the rate of 150 mg per kg diet displayed higher final body weight than the control (*p* < .05) (Table 2). Furthermore, rabbits fed with *A. awamori*‐enriched diets at 50, 100 and 150 mg per kg diet showed significantly higher weight gain than the control (*p* < .05). The feed conversion ratio was decreased in rabbits fed *A. awamori* at 100 mg/kg followed by the level of 50 mg/kg; however, the differences from rabbits fed 150 mg per kg were not apparent with the other *A. awamori*‐enriched diets. No significant differences were observed between the feed intake of control rabbits and those fed with enriched diets (*p* > .05).

**TABLE 2:** Growth performance and feed utilization of rabbits fed varying levels of *Aspergillus awamori* *

Item	*Aspergillus awamori* (mg/kg diet)				*p* value
	0	50	100	150	
Initial body weight (g)	850.00 ± 14.10	860.00 ± 15.00	850.00 ± 17.00	860.00 ± 16.00	.20
Final body weight (g)	2,270.00 ± 47.00^a^	2,460.00 ± 60.12^a^	2,490.00 ± 7.00^ab^	2,730.00 ± 2.00^b^	.02
Weight gain (g)	1,420.00 ± 7.00^a^	1,590.00 ± 36.05^b^	1,680.00 ± 3.00^b^	1,870.00 ± 4.40^b^	.01
Feed intake (g)	4,230.00 ± 22.00	4,390.00 ± 19.06	4,280.00 ± 9.00	5,303.00 ± 11.00	.35
Feed conversion ratio	2.99 ± 0.06^c^	2.75 ± 0.04^b^	2.55 ± 0.03^a^	2.84 ± 0.05^ab^	.01

* Values expressed as means ± *SEM*. Different superscript letters indicate significant differences (*p* < .05).

### 3.2 Digestibility coefficients and intestinal morphometry

**FIGURE 1:**
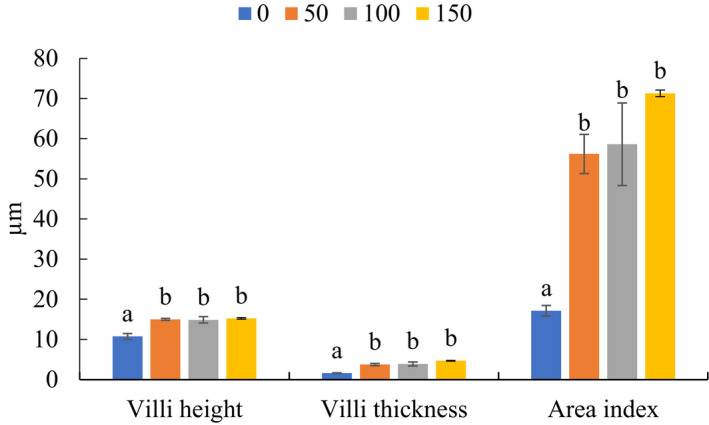
Intestinal villi measurements of rabbits fed varying levels of *Aspergillus awamori*. Values expressed as means ± *SEM*. Different superscript letters indicate significant differences (*p* < .05)

Rabbits fed with *A. awamori*‐enriched diets at 100 or 150 mg per kg diet had significantly higher protein, lipid and fibre digestibility coefficients relative to control animals (*p* < .05) (Table 3). Digestibility coefficients for organic matter and dry matter were, however, not affected by *A. awamori* supplementation (*p* > .05). Rabbits fed varying levels of *A. awamori* also had significantly higher intestinal villi measurements (height and thickness) (*P* ˂ 0.05) (Figure 1).

**TABLE 3:** Digestibility coefficients (%) of rabbits fed varying levels of *Aspergillus awamori* *

Item	*Aspergillus awamori* (mg/kg diet)				*p* value
	0	50	100	150	
Crude protein	73.22 ± 1.06^a^	74.27 ± 0.74^a^	78.11 ± 1.25^b^	79.23 ± 1.04^b^	.001
Total lipids	76.22 ± 1.25^a^	78.46 ± 1.11^a^	79.82 ± 1.22^b^	80.21 ± 1.18^b^	.02
Crude fibres	36.15 ± 2.13^a^	37.15 ± 0.84^a^	41.22 ± 2.54^b^	43.23 ± 2.15^b^	.04
Dry matter	66.21 ± 1.02	68.58 ± 1.31	70.11 ± 1.22	72.73 ± 2.04	.31
Organic matter	64.28 ± 1.45	65.34 ± 1.62	66.11 ± 1.11	67.23 ± 1.86	.24

* Values expressed as means ± *SEM*. Different superscript letters indicate significant differences (*p* < .05).

### 3.3 Blood indices

The blood indices of rabbits fed *A. awamori* showed no significant differences with the exception of RBC, haemoglobin, glucose and total protein (Tables 4 and 5). RBC and haemoglobin increased in rabbits fed with 150 mg *A. awamori* per kg diet (*P* ˂ 0.05), as did blood total protein (Table 5). Notably, the serum glucose content was significantly decreased in rabbits fed *A. awamori* at the rate of 50, 100 and 150 mg per kg diet (*P* ˂ 0.05) (Table 5).

**TABLE 4:** Blood haematology parameters of rabbits fed varying levels of *Aspergillus awamori* *

Item	*Aspergillus awamori* (mg/kg diet)				*p* value
	0	50	100	150	
RBCs (×10^6^/µl)	4.23 ± 0.24^a^	4.30 ± 0.00^a^	4.67 ± 0.03^ab^	4.85 ± 0.10^b^	.03
Haemoglobin (g/dl)	8.20 ± 0.30^a^	8.53 ± 0.11^ab^	8.64 ± 0.15^ab^	8.73 ± 0.07^b^	.03
PCV (%)	31.67 ± 0.89	33.00 ± 0.58	32.67 ± 0.67	31.67 ± 0.33	.72
MCV (fl)	74.87 ± 3.23	76.74 ± 2.88	69.96 ± 3.11	65.30 ± 2.14	.52
MCH (pg)	19.39 ± 0.87	19.84 ± 0.91	18.50 ± 0.66	18.00 ± 0.49	.43
MCHC (%)	25.89 ± 1.22	25.85 ± 1.76	26.45 ± 1.45	27.57 ± 0.89	.06
WBCs (×10^3^/µl)	7.87 ± 0.20	8.63 ± 0.64	8.87 ± 0.58	8.77 ± 0.29	.06
Lymphocyte (%)	54.33 ± 0.67	63.00 ± 1.00	64.33 ± 2.91	59.67 ± 0.33	.12
Heterotrophil (%)	38.33 ± 1.33	29.67 ± 1.45	28.00 ± 3.21	33.00 ± 3.00	.23
Monocyte (%)	5.67 ± 0.88	5.67 ± 0.33	6.00 ± 0.00	5.67 ± 0.33	.62
Eosinophil (%)	1.67 ± 0.33	1.67 ± 0.33	1.67 ± 0.33	1.67 ± 0.33	.22
H/L	0.66 ± 0.02	0.47 ± 0.02	0.44 ± 0.07	0.55 ± 0.05	.11

Abbreviations: MCH, mean cell haemoglobin; MCHC, mean corpuscular haemoglobin concentration; MCV, Mean corpuscular volume; PCV, packed cell volume; RBCs, red blood cells; WBCs, white blood cells.

* Values expressed as means ± *SEM*. Different superscript letters indicate significant differences (*p* < .05).

**TABLE 5:** Blood biochemical parameters of rabbits fed varying levels of *Aspergillus awamori* *

Item	*Aspergillus awamori* (mg/kg diet)				*p* value
	0	50	100	150	
Total protein (mg/dl)	8.23 ± 0.09^a^	8.20 ± 0.15^a^	8.57 ± 0.18^ab^	9.12 ± 0.26^b^	.02
Albumin (g/dl)	4.03 ± 0.03	3.97 ± 0.03	4.00 ± 0.06	3.90 ± 0.06	.13
Globulin (g/dl)	4.20 ± 0.12	4.23 ± 0.17	4.57 ± 0.24	4.57 ± 0.29	.06
A/G	0.96 ± 0.04	0.94 ± 0.04	0.88 ± 0.06	0.86 ± 0.07	.22
Glucose (mg/dl)	92.33 ± 5.81^b^	79.67 ± 0.67^a^	77.67 ± 3.93^a^	73.67 ± 2.73^a^	.03
AST (U/I)	54.00 ± 3.61	50.33 ± 2.90	51.67 ± 1.20	54.33 ± 2.19	.31
ALT (U/I)	17.33 ± 2.73	15.33 ± 2.03	16.00 ± 2.08	19.67 ± 2.40	.32
Urea (mg/dl)	39.33 ± 0.67	38.67 ± 0.67	38.20 ± 0.42	41.00 ± 1.00	.12
Creatinine (mg/dl)	0.87 ± 0.03	0.83 ± 0.03	0.80 ± 0.06	0.97 ± 0.09	.32

Abbreviations: A/G, albumin‐to‐globulin ratio; AST, aspartate aminotransferase; ALT, alanine aminotransferase.

* Values expressed as means ± *SEM*. Different superscript letters indicate significant differences (*p* < .05).

### 3.4 Immune response

**FIGURE 2:**
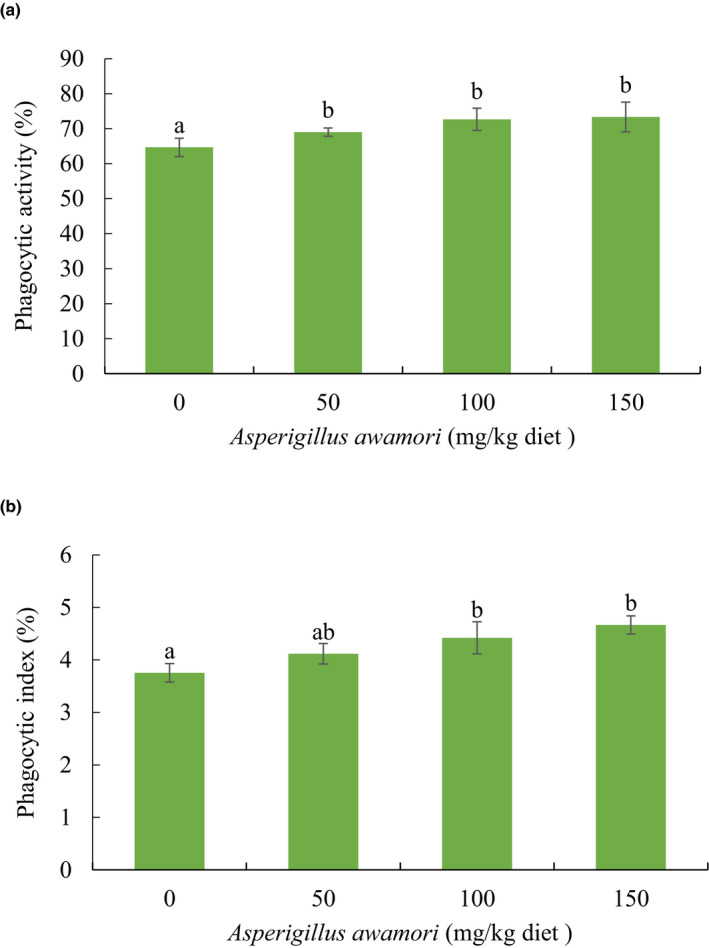
(a) Phagocytic activity and (b) phagocytic index of rabbits fed varying levels of *Aspergillus awamori*. Values expressed as means ± *SEM*. Different superscript letters indicate significant differences (*p* < .05)

Animals fed with all three *A. awamori‐*enriched diets presented significantly higher phagocytic activity (*p* < .05) (Figure 2a). Additionally, the phagocytic index was significantly higher in rabbits fed with 100 mg or 150 mg (*p* < .05), but not 50 mg (*p* > .05), *A. awamori* per kg diet relative to the control (Figure 2b).

### 3.5 Oxidative status

**FIGURE 3:**
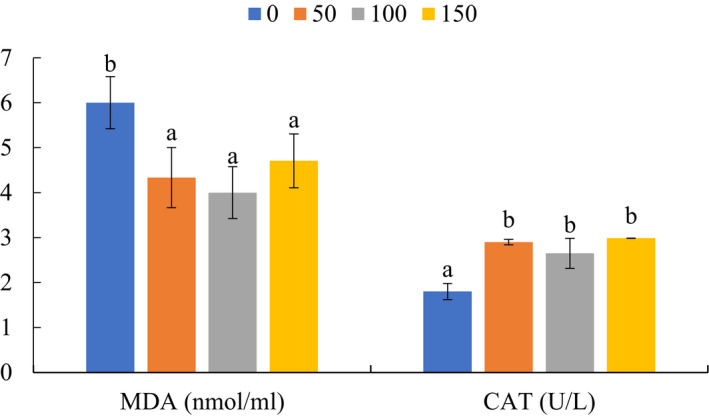
Malondialdehyde (MDA, nmol/ml) and catalase activity (CAT, U/L) of rabbits fed varying levels of *Aspergillus awamori*. Values expressed as means ± *SEM*. Different superscript letters indicate significant differences (*p* < .05)

The concentration of MDA and CAT activity of rabbits fed varying levels of *A. awamori* are presented in Figure 3. Compared with the control group, CAT activity increased and MDA level decreased in rabbits fed with *A. awamori* regardless of the dose (*P* ˂ 0.05).

## 4 DISCUSSION

Probiotics have attracted the attention of nutritionists because of their beneficial influence on animals; they can improve innate immune response, growth and feed digestibility (Saleh, Amber, et al., 2014; Saleh et al., 2012). In the present study, for the first time, we reported that dietary *A. awamori* improved the growth, intestinal morphology, immune responses and antioxidative responses of growing rabbits.

Here, supplementation with *A. awamori* continuously for 8 weeks positively increased the growth performance of rabbits. Probiotic feeding may have increased growth through the secretion of exogenous enzymes in the gastrointestinal tract that can facilitate the digestion and absorption of food and nutrients (Copeland et al., 2009; Kritas et al., 2008). Thus, improved growth performance in growing rabbits fed with *A. awamori* is likely linked to improvements in their digestive system and higher feeding efficiency (Bhatt et al., 2017; Fuller, 1989; Phuoc & Jamikorn, 2017).

In the current study, the digestibility coefficients of APRI rabbits confirmed the beneficial role of *Aspergillus* in feed digestion. Indeed, protein, lipid and fibre digestibility coefficients were enhanced by *A. awamori* supplementation; this finding is in agreement with previous studies that reported increased digestibility coefficients in rabbits fed with probiotics (Bhatt et al., 2017; Phuoc & Jamikorn, 2017). The possible reason for the enhanced digestibility coefficients is associated with the potential role of *A. awamori* to facilitate the digestion of fibres and protein through the secretion of cellulase, xylanase and protease (Saleh, Hayashi, Ijiri, & Ohtsuka, 2014). Furthermore, *Aspergillus* can degrade the antitrypsin and antinutritional factors which are presented in soybean meal and the other plant originated ingredients and in turn increase the protein and lipids absorption in the gut (de Castro, Castilho, & Freire, 2015; Yamamoto, Saleh, Ohtsuka, & Hayashi, 2005). In parallel with the current study, Saleh, Amber, et al., 2014 reported that broilers fed diets with *A. awamori* displayed increased protein, lipid and fibre digestibility coefficients. The increased digestibility of lipids is correlated with influence of *A. awamori* on increasing the digestibility of non‐digestible carbohydrates and give rise to short‐chain fatty acids which can facilitate the absorption of the nutrients through the intestinal epithelial cells (Simonová, Lauková, Žitňan, & Chrastinová, 2015).

The intestinal morphometrical analysis was normally measured during the evaluation of using probiotics which presents a clear pathway for the digestion and absorption process of nutrients (Mourão et al., 2006; Seyidoglu & Peker, 2015). The inclusion of *A. awamori* in growing rabbits’ diet resulted in improved intestinal length and villi thickness. Thus, improved feed utilization can be attributed to the increased absorptive area provided by the increased villi length and thickness (Peker, SEYİDOĞLU, N., Galip, N., & Zik, B., 2014). The increased villus length an thickness obtained in the present study could be due to increased epithelial cell turnover and absorption of nutrients (Oso et al., 2013). Probiotics not only increase the production of metabolites such as enzymes and antimicrobial substances but they are also beneficial for enhancing the absorption capability of the intestinal barriers (Machida et al., 2005). Furthermore, probiotics can inhabit in the intestines of animals and help the digestion process according to their type, dose and period of administration (Fathi et al., 2017).

The haematological and serum biochemistry parameters measured here were rarely altered by *A. awamori*; this suggests that probiotics may be safe for use due to their low ability to trigger adverse changes in the blood and the liver. However, red blood cells and haemoglobin levels did increase with *A. awamori* supplementation relative to the control, which may indicate that the probiotic stimulated the rabbits’ immune system as supplementation levels increased. Our findings are similar to those of Fathi et al. (2017), who reported that dietary probiotics increased haemoglobin levels and RBC in rabbits.

Cortisol and glucose levels can be used as an efficient indicator of stressful conditions (Illera, González Gil, Silván, & Illera, 2000). In addition, cortisol regulates the plasma ionic content, and oxidative and immune responses through the production of glucose, which controls the level of energy in animals’ cell (Ledbetter & Lippert, 2002). The reduced levels of glucose observed in the present study may indicate the safe and beneficial effects of *A. awamori* for growing rabbits.

Serum total proteins, as the most significant components of blood serum, are thought to be effective indicators of humoral immunity and well‐being in rabbits (Guo et al., 2017). In our study, dietary *A. awamori* supplementation elevated the level of total serum protein, which could indicate the improved immunity of rabbits (El‐Katcha, Ismail, Soltan, & El‐Naggar, 2011). The increased total serum protein indicates that the protein content of the diets was adequate and available and that *A. awamori* inclusion levels increased the protein metabolism and synthesis through the activation of protease (Olorunsola et al., 2016).

Phagocytic cells can produce antimicrobial substances, such as lysosomal enzymes, which are vital tools for attacking infectious diseases (Abdelnour et al., 2018). In relation to their immune response, growing rabbits fed *A. awamori* exhibited a significant increase in phagocytic activity and in their phagocytic index compared with control rabbits. Similarly, El‐Katcha et al. (2011) reported that rabbits showed improved phagocytosis after probiotic feeding. The increased phagocytosis exhibited by growing rabbits in the current study might be due to their increased immunity and antioxidant capacity following *A. awamori* feeding.

An oxidative emphasis normally occurs when the creation and elimination of free radicals (ROS) are unbalanced as the oxidative damage of cultured species is directly related to the quality of diet (Apel & Hirt, 2004; Winiarska, Fraczyk, Malinska, Drozak, & Bryla, 2006). Antioxidant enzymes such as superoxide dismutase, glutathione peroxidase and CAT are important scavengers of ROS; they protect the body tissues from oxidative stress damage (Gaetani et al., 1996). MDA levels are a product of lipid peroxides and high levels of ROS, which can cause damage to the cell's DNA, protein and cytoplasm (Wang et al., 2018; Yao et al., 2010). In the present study, we observed that rabbits fed *A. awamori* showed enhanced CAT with reduced MDA; this confirms that the known antioxidant properties of this probiotic are not lost when administered orally in growing rabbits. Similarly, earlier reports revealed that probiotic feeding increased CAT while reducing MDA levels (Solis de los Santos et al., 2005). The mechanism of dietary *A. awamori* in decreasing the MDA content remains unclear, however, the possible explanation is associated with the activity of the antioxidant substances produced by *A. awamori* (Bhanja, Kumari, & Banerjee, 2009). Kanauchi et al. (2008) reported that *A. awamori* produces a natural material called feruloyl esterase which acts as antioxidative substances. In line with the present study, Abdelhady et al. (2017) reported that *A. awamori* generated increased antioxidative status in rabbits through upregulating the antioxidative‐related genes.

## 5 CONCLUSION

Based on our data on rabbit growth, feed efficiency, intestine histology and nutrient digestibility, *A. awamori* appears to provide benefits to growing rabbits’ performance without any indications of negative physiological impacts. Indeed, the addition of *Aspergillus* to their diet reduced the damaging effects of oxidative stress and improved the rabbits’ immune status. Thus, *A. awamori* can be considered as a probiotic candidate for use with growing rabbits. Specifically, we recommend that 100–150 mg *A. awamori* per kg of rabbit feed is applied to the diet of growing rabbits as a feed supplement.

## CONFLICT OF INTEREST

The authors have declared no conflict of interest.

## AUTHOR CONTRIBUTION

Mahmoud H. El‐Deep: Conceptualization; Data curation; Formal analysis; Investigation; Methodology. Mahmoud Dawood: Conceptualization; Data curation; Formal analysis; Funding acquisition; Investigation; Methodology; Visualization; Writing‐original draft; Writing‐review & editing. Mohamed H. Assar: Conceptualization; Formal analysis; Investigation; Methodology; Supervision. Bilal Ahamad Paray: Conceptualization; Funding acquisition; Methodology; Visualization; Writing‐original draft; Writing‐review & editing.

### PEER REVIEW

The peer review history for this article is available at https://publons.com/publon/10.1002/vms3.345.
